# Disentangling the neurological basis of chronic ocular pain using clinical, self-report, and brain imaging data: use of K-means clustering to explore patient phenotypes

**DOI:** 10.3389/fneur.2023.1265082

**Published:** 2023-11-16

**Authors:** Scott Holmes, Nicholas Reyes, Jaxon J. Huang, Anat Galor, Pradip M. Pattany, Elizabeth R. Felix, Eric A. Moulton

**Affiliations:** ^1^Pain and Affective Neuroscience Center, Department of Anesthesia, Critical Care and Pain Medicine, Boston Children’s Hospital, Harvard Medical School, Boston, MA, United States; ^2^Pediatric Pain Pathway Lab, Boston Children’s Hospital, Harvard Medical School, Boston, MA, United States; ^3^Surgical Services, Miami Veterans Administration Medical Center, Miami, FL, United States; ^4^Bascom Palmer Eye Institute, University of Miami, Miami, FL, United States; ^5^Department of Radiology, University of Miami, Miami, FL, United States; ^6^Research Service, Miami Veterans Administration Medical Center, Miami, FL, United States; ^7^Physical Medicine and Rehabilitation, University of Miami, Miami, FL, United States; ^8^Brain and Eye Pain Imaging Lab, Department of Anesthesia, Critical Care and Pain Medicine, Boston Children’s Hospital, Harvard Medical School, Boston, MA, United States; ^9^Department of Ophthalmology, Boston Children’s Hospital, Harvard Medical School, Boston, MA, United States

**Keywords:** ocular pain, functional MRI, chronic pain, pain mechanism, pain behavior

## Abstract

**Introduction:**

The factors that mediate the expression of ocular pain and the mechanisms that promote chronic ocular pain symptoms are poorly understood. Central nervous system involvement has been postulated based on observations of pain out of proportion to nociceptive stimuli in some individuals. This investigation focused on understanding functional connectivity between brain regions implicated in chronic pain in persons reporting ocular pain symptoms.

**Methods:**

We recruited a total of 53 persons divided into two cohorts: persons who reported no ocular pain, and persons who reported chronic ocular pain, irrespective of ocular surface findings. We performed a resting state fMRI investigation that was focused on subcortical brain structures including the trigeminal nucleus and performed a brief battery of ophthalmological examinations.

**Results:**

Persons in the pain cohort reported higher levels of pain symptoms relating to neuropathic pain and ocular surface disease, as well as more abnormal tear metrics (stability and tear production). Functional connectivity analysis between groups evinced multiple connections exemplifying both increases and decreases in connectivity including regions such as the trigeminal nucleus, amygdala, and sub-regions of the thalamus. Exploratory analysis of the pain cohort integrating clinical and brain function metrics highlighted subpopulations that showed unique phenotypes providing insight into pain mechanisms.

**Discussion:**

Study findings support centralized involvement in those reporting ocular-based pain and allude to mechanisms through which pain treatment services may be directed in future research.

## Introduction

A lack of knowledge surrounding the mechanisms that predispose an individual to develop chronic ocular pain are limiting the ability and efficacy of clinical intervention. Ocular surface diseases, including dry eye (DE) and Meibomian gland dysfunction (MGD), are highly prevalent conditions in the general population, and can range from mild to severe (1), with the latter category leading to significant pain and decreased quality of life (2, 3). However, not all individuals with ocular pain have notable ocular surface abnormalities and not all individuals with ocular surface abnormalities report pain. The basis from which some people develop pain-related symptoms and others are pain-free is unclear as pain complaints are often, but not always, discordant from objective metrics of ocular pathology (4–6). One hypothesis is that central mechanisms underlie ocular pain conditions in some individuals. The paucity of literature on this topic points to an immediate need to understand the contribution of central mechanisms associated with ocular pain.

Chronic ocular pain can overlap with reports of altered mental health and behavioral abnormalities. For example, persons with a diagnosis of dry eye disease may show elevated levels of depression and anxiety—more prevalent in severe dry eye conditions—with *symptoms* correlating with mental health indices more so than *signs* (7, 8). Patients who report high levels of neuropathic ocular pain also report lower quality of life and increased mental health scores than persons with low levels of neuropathic ocular pain (9). Notably, symptoms of neuropathic ocular pain are often discordant from objective pathology (10–12) suggesting a centralized, or sub-clinical component that is not currently being recognized. As such, there needs to be considerations beyond the ocular surface to more fully understand chronic ocular pain mechanisms and the impact of this condition on health and function.

Central nervous system changes in persons with ocular pain have a complex relationship with self-reported pain and pain behaviors. This is true both in cases of ocular pain driven primarily by nociceptive mechanisms and those driven primarily by neuropathic mechanisms. For example, in a case report of a person with *acute* pain due to a corneal epithelial abrasion (e.g., nociceptive mechanism), increased activation of the trigeminal nucleus (as well as trigeminal ganglion) was found alongside reports of photophobia/hypersensitivity to light (13). Prior work using fMRI in persons with *chronic* ocular surface pain with neuropathic features has shown that subjective pain reports correlated with light-evoked activation in brain areas including the trigeminal nucleus, primary somatosensory cortex, anterior midcingulate cortex, and insula (14). Beyond stimulus-evoked activation, other studies have focused on central connectively at rest in individuals with ocular pain. In persons with pain due to corneal ulcers (e.g., nociceptive mechanism), higher degree centrality—a metric of connectivity—was found in diverse cortical regions (e.g., frontal lobe, precuneus, inferior parietal lobule, posterior cingulate, occipital lobe, and temporal lobe) relative to healthy controls (15). Interestingly, central changes have also been noted in individuals with a diagnosis of dry eye disease in relation to light sensitivity, a form of ocular pain. Task-based fMRI using a light stimulation paradigm in persons with dry eye (defined through clinical examination, oxford scores, tear break up time, and Schirmer tests) and light sensitivity has shown greater activation in the occipital cortex and less deactivation of the superior temporal cortex in patients relative to healthy controls (16). These previous studies suggest that individuals with ocular pain from multiple etiologies exhibit functional changes at multiple levels including the trigeminal circuitry and higher-order central nervous system components as well.

The complex projections emanating from trigeminal nerve afferents through brainstem and sub-cortical regions support an investigation into the primary checkpoints of afferent sensory signaling in the brain. That is, prior work has established primary somatosensory and motor areas, as well as amygdala, lateral prefrontal cortex, presupplementary motor area, basal ganglia, cerebellum, and brainstem as core regions (17) (for review see (18)). A direct connection between the periaqueductal gray (PAG) and the trigeminal brainstem nuclei (19, 20) has been observed, linking PAG activity as a potential form of pain modulation to the trigeminal sensory complex. Lastly, the thalamus plays a critical role mediating the relay of nociceptive stimuli through the cortex to support the perception of pain (10, 21, 22). Based on this need, the aims of this hypothesis-generating investigation were to (1) evaluate the extent to which pain behaviors are differentially reported between cohorts with and without chronic ocular pain, (2) evaluate functional brain network alterations relating to chronic ocular pain, and (3) explore the extent to which integrating neuroimaging and clinical data could provide insight into chronic ocular pain sub-cohorts.

## Methods

### Standard protocol approvals, registrations, and patient consents

The study was approved by the Miami Veterans Affairs (VA) and the University of Miami Institution Review Boards (IRB approvals #3011.08 and 20190340, respectively). The study was conducted in accordance with the principles of the Declaration of Helsinki and complied with the requirements of the United States Health Insurance Portability and Accountability Act. Written informed consent was obtained from all participants prior to any study activities.

### Study population

We recruited 53 subjects who presented to the Miami VA eye clinic for yearly screening and divided them into two groups: patients with current ocular surface pain (rating of average ocular pain over 1 week recall ≥1, scale 0–10) and who reported having ocular pain that began at least 3 months prior (i.e., chronic eye pain, *n* = 37) and patients without pain (rating of average ocular pain over 1 week recall = 0, *n* = 16). Exclusion criteria for both groups included ocular diseases that could confound pain, such as glaucoma; use of glaucoma medications; uveitis; iris transillumination defects; retinal degeneration; and anatomic abnormalities of the cornea, conjunctiva, or eyelids. We also excluded individuals with contraindications to fMRI scanning (e.g., pregnancy, pacemaker, and implanted metal device).

### Questionnaires

Subjects were administered questionnaires to collect demographic and health information, including age, sex, race, ethnicity, and medical history. Standardized DE questionnaires included the Dry Eye Questionnaire 5 (DEQ-5) (23) and Ocular Surface Disease Index (OSDI) (24). The Neuropathic Pain Symptom Inventory-modified for the Eye (NPSI-Eye), a validated eye-centric variation of the NPSI (25), was obtained to quantify neuropathic-like eye pain symptoms. We also included two items from the Visual Light Sensitivity Questionnaire (VLSQ) (26) pertaining to “In the past month, how often did you have visual light sensitivity outdoors during daylight?” (Q1) and “In the past month, how often did you need to wear dark glasses on cloudy days or indoors?” (Q7). Both items were rated on a five-point scale including: Never, Rarely, Sometimes, Often, and Always.

### Ocular surface evaluation

Each patient underwent a clinical exam that included (in the order performed) tear breakup time (TBUT, measured in s; lower values indicate less tear stability), corneal staining [graded to the National Eye Institute scale (27); higher values indicate more epithelial irregularity], and anesthetized tear production using Schirmer strips (measured by mm of wetting at 5 min; lower values indicate lower tear production).

### Quantitative sensory testing

All QST was performed on the right ventral forearm, at the midpoint between the wrist and cubital fossa. This test site was chosen as it is both remote from the eye, allowing for assessment of systemic somatosensory function absent of peripheral mechanisms at the affected site, and it is a commonly used test site across studies (2). All QST stimuli were delivered using a Medoc TSA-II device with a 30 mm × 30 mm thermal contact probe. Report of painful aftersensations were separately captured after two protocols of thermal noxious stimulation: (1) repeated presentation of a cold stimulus, set at 6.3°C (2°C below the average cold pain threshold for 20 participants in a cohort group from the same patient population [citation]): and (2) repeated presentation of a hot stimulus, set at 45.5°C (1°C above the average hot pain threshold from this same group of 20 participants). For each stimulus series, the thermode was set to the prescribed temperature and 10 1 s stimulus presentations were manually delivered to the skin at a rate of 0.5 Hz. At the end of the last stimulus presentation, a timer was started to mark the 30-s post-stimulus time point. At this time point, the participant was asked to rate the present intensity of pain at the site of testing (i.e., “aftersensation”). The cold pain aftersensation trial was conducted first, with a minimum of 3 min rest before the first stimulus of the hot pain aftersensation trial began. The presence of any 30-s aftersensations (pain intensity rating > 0) on the forearm was recorded.

### fMRI acquisition and preprocessing

Imaging was conducted using a 3 T Siemens MAGNETOM Vida scanner (Erlangen, Germany) with a BioMatrix Head/Neck 20 channel coil. For anatomical scans, a sagittal three-dimensional T1-weighted scan (MPRAGE) was performed [TE/TR = 2.38/2,100 ms; 192 1.00 mm-thick sagittal slices; in-plane resolution = 1.00 mm × 1.00 mm (256 × 256)]. For the functional scan, a gradient echo (GE) echo planar imaging (EPI) sequence was performed [TE/TR = 30/2,000 ms; 100 1.50 mm-thick oblique slices aligned to the long axis of the caudal brainstem; in-plane resolution = 1.94 mm × 1.94 mm (136 × 136)], with 303 volumes (10 min and 6 s) captured. The oblique orientation of acquisition has proven useful for functional imaging of brainstem structures (15).

### Image processing

Functional neuroimaging data were processed using the CONN toolbox (28). Data were processed using the standard pipeline that included: realignment and unwarping (motion estimation and correction), slice-timing correction, outlier detection, segmentation (CSF and white matter) and normalization, smoothing with an 8 mm filter. Confounding effects were addressed including from white matter, CSF, realignment, scrubbing, and effects of rest and were applied prior to band-pass filtering. The resulting images were processed using a band-pass filter with the following range: 0.008 and 0.09. Our field of view was focused on improved resolution of brainstem and sub-cortical structures. Regions of interest included the thalamus, caudate, putamen, pallidum, amygdala, accumbens, as well as the brainstem, periaqueductal gray, and trigeminal nucleus (29, 30). Based on the extensive inter-connectivity of the thalamus, we included sub-regions of the thalamus outlined in Akram et al. (31). We also included bilateral precentral and postcentral gyrus as prior work has documented changes in these regions in persons with chronic pain (32) and they were available within our restricted field of view.

### Exploratory analysis

We performed a K-means clustering analysis on our chronic ocular pain cohort (*n* = 37) to separate these participants. We use silhouette coefficients to determine the number of clusters to use in our pain sub-cohorts. All clinical data were included as well as the output functional connectivity matrices from all 27 regions. We performed a principal component analysis on the functional connectivity matrices from each participant and extracted the top 20 components that represented 80% of the variance in the data. We chose two clusters based on the limited sample size of the starting population. The use of K-means clustering was performed using the Sklearn (33) python package after transforming all participant data into *z*-scores. We also performed the SelectKBest package to understand the feature performance in differentiating the two pain cohorts and then back projected cluster data into original clinical metrics (not in *z*-score format) for clinical interpretation.

### Statistical analysis

This was a hypothesis generating study. All clinical and demographic data were compared between cohorts using *t*-tests when data were normally distributed and Mann–Whitney tests when data were not normally distributed. Sex-distribution between cohorts was performed using Chi-square tests. Functional connectivity analysis was performed using a two-sided connectivity threshold of *p* = 0.05 (uncorrected).

## Results

### Demographic and clinical data

A summary of demographic, self-reported comorbidities, and self-reported medications is presented in Table 1. No significant group differences in age, or distribution of ethnicity or race, self-reported comorbidities, or self-reported medications were detected between the no-pain and the pain groups. A total of 31 and nine participants had a dry eye diagnosis from the pain and no-pain cohorts, respectively. Participants from the two cohorts were found to differ based on responses to self-report questionnaires related to DE symptoms and ocular pain severity and some tear parameters (TBUT and Schirmers) but were no different in terms of years of DE diagnosis, or corneal staining (Table 2). The presence of unpleasant aftersensations was reported more frequently by individuals in the pain cohort (16/37) compared to the no-pain cohort (2/16).

**Table 1:** Demographics and co-morbidities of subjects.

	Pain (*n* = 37)	No-pain (*n* = 16)	*p* value
Demographics			
Age (mean ± SD; years)	55.9 ± 10.7	54.8 ± 9.3	0.71
Sex, male % (*n*)	76% (28)	63% (10)	0.18
Race, White % (*n*)	92% (34)	69% (11)	0.34
Ethnicity, Hispanic % (*n*)	51% (19)	50% (8)	1.0
Self-reported comorbidities			
Diabetes mellitus % (*n*)	3% (1)	13% (2)	0.21
PTSD % (*n*)	41% (15)	31% (5)	0.52
Depression % (*n*)	65% (24)	63% (10)	0.87
Arthritis % (*n*)	32% (12)	19% (3)	0.51
Sleep apnea % (*n*)	49% (18)	44% (7)	0.98
Migraine % (*n*)	35% (10)	13% (2)	0.30
Traumatic brain injury % (*n*)	16% (6)	6% (1)	0.66
Past or current smoker % (*n*)	46% (18)	69% (11)	0.38
Self-reported medications			
Antidepressants % (*n*)	51% (19)	38% (6)	0.27
Anxiolytics % (*n*)	46% (17)	38% (6)	0.79
Gabapentin % (*n*)	19% (7)	13% (2)	0.71
Pregabalin % (*n*)	3% (1)	0% (0)	1.0
NSAIDs % (*n*)	32% (12)	25% (4)	0.75

*n*, Number of subjects; SD, Standard deviation; PTSD, Post-traumatic stress disorder; NSAIDs, Non-steroidal anti-inflammatory drugs. ^‡^Chi-squared test, *p* < 0.05.

**Table 2:** Ocular symptoms and signs of subjects.

	Cases (*n* = 37)	Controls (*n* = 16)	*p* value
DE, light sensitivity, and ocular pain symptoms assessed via questionnaires, mean ± SD (*n*)			
Years of DE diagnosis, mean ± SD (*n*)	9.2 ± 6.8 (31)	8.4 ± 11.5 (9)	0.88
DEQ5 (range 0–22), mean ± SD (*n*)	14.2 ± 3.6 (37)	4.5 ± 3.9 (16)	<0.01^*^
OSDI-Q1 (range 0–4), mean ± SD (*n*)	2.9 ± 1.2 (37)	0.7 ± 1.1 (15)	<0.01^*^
VLSQ-8-Q1 (range 0–4), mean ± SD (*n*)	4.0 ± 0.9 (35)	1.3 ± 1.0 (16)	<0.01^*^
VLSQ-8-Q7 (range 0–4), mean ± SD (*n*)	3.4 ± 1.0 (35)	1.1 ± 1.1 (16)	<0.01^*^
NPSI-Eye-Q9 (pain provoked by light) (range 0–10), mean ± SD (*n*)	6.9 ± 5.0 (37)	0.0 ± 0.0 (16)	<0.01^*^
NPSI-Eye total (range 0–100), mean ± SD (*n*)	33.5 ± 19.4 (35)	1.1 ± 1.9 (15)	<0.01^*^
OSDI total (range 0–100), mean ± SD (*n*)	56.6 ± 21.6 (37)	9.9 ± 12.3 (15)	<0.01^*^
Rating of average eye pain during the past week (range 0–10), mean ± SD (*n*)	4.9 ± 2.6 (37)	0.0 ± 0.0 (16)	<0.01^*^
Rating of average non-ocular pain during the past week (range 0–10), mean ± SD (*n*)	5.3 ± 2.7 (35)	2.3 ± 2.5 (16)	<0.01^*^
Ocular pain before and after anesthesia			
Ocular pain rating before anesthesia (mean ± SD; range 0–10) (*n*)	3.4 ± 2.8 (36)	0.2 ± 0.5 (15)	<0.01^*^
Ocular pain rating after anesthesia (mean ± SD; range 0–10) (*n*)	2.5 ± 2.7 (36)	0.2 ± 0.6 (15)	<0.01^*^
Tear parameters			
TBUT OD (mean ± SD; s) (*n*)	7.0 ± 3.7 (36)	10.8 ± 3.7 (15)	<0.01^*^
TBUT OS (mean ± SD; s) (*n*)	8.1 ± 4.0 (36)	10.8 ± 4.6 (15)	0.57
Staining OD (mean ± SD; range 0–15) (*n*)	2.3 ± 2.3 (36)	2.3 ± 4.3 (15)	0.90
Staining OS (mean ± SD; range 0–15) (*n*)	2.0 ± 2.2 (36)	1.7 ± 3.0 (15)	0.56
Schirmer’s OD (mean ± SD; mm) (*n*)	11.8 ± 9.1 (36)	19.1 ± 10.3 (15)	0.03^*^
Schirmer’s OS (mean ± SD; mm) (*n*)	12.0 ± 9.4 (36)	18.2 ± 9.8 (15)	0.05^*^

DE, Dry eye; SD, Standard deviation; *n*, Number of subjects; DEQ5, 5-item dry eye questionnaire; OSDI, Ocular surface disease index; OSDI-Q1, Ocular surface disease index question #1: Have you experienced eyes that are sensitive to light during the past 1 week?; VLSQ-8, Visual light sensitivity questionnaire-8; VLSQ-8-Q1, Visual light sensitivity questionnaire-8 question #1: In the past month, how often did you have visual light sensitivity to outdoors during daylight?; VLSQ-8-Q1, Visual light sensitivity questionnaire-8 question #7: How often does sensitivity to light limit your ability to read, watch TV, or use the computer?; NPSI-Eye, Neuropathic pain symptom inventory modified for the eye; NPSI-Eye-Q9, Neuropathic pain symptom inventory modified for the eye question #9: During the past 24 h, if your eye pain provoked or increased by light?; TBUT, Tear break-up time. *Independent *t*-test, *p* < 0.05.

### Brain imaging

**Figure 1:**
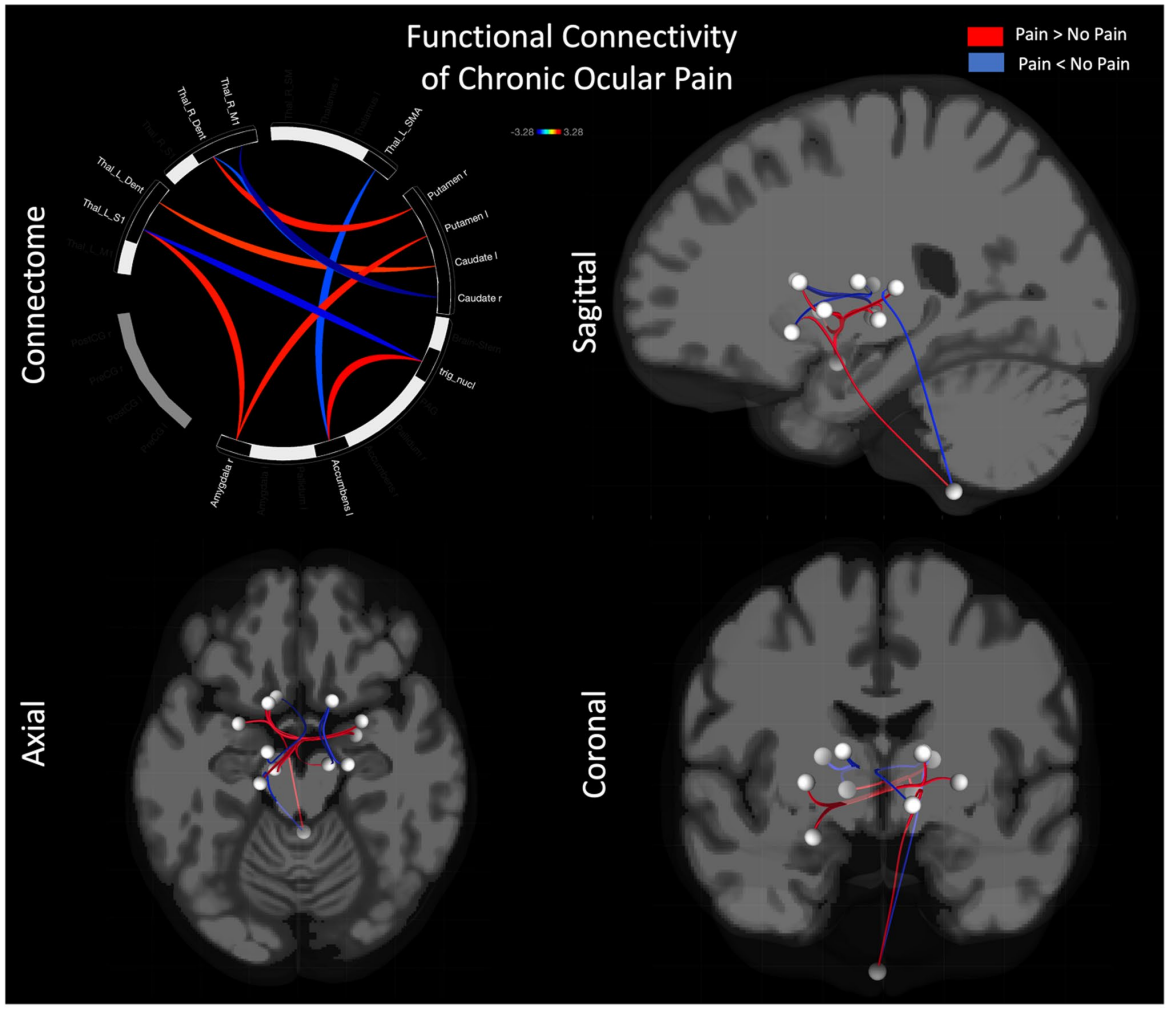
Functional connectivity differences between pain and no-pain cohorts. Differences in functional connectivity are shown using a connectome (i) as well as glass brain in sagittal (ii), axial (iii), and coronal (iv) views.

Performing resting state functional connectivity between cohorts showed both increased and decreased functional connectivity differences (Figure 1; Table 3). Main regions showing group differences were found in bilateral sub-cortical areas including amygdala, accumbens, thalamus, caudate, and putamen. Multiple significant findings were found in sub-regions of the thalamus including the M1, S1, SMA, and dentate regions of the thalamus. No significant findings were observed in the pre-or post-central gyrus.

**Table 3:** Outcomes from the functional connectivity analysis.

Brain regions			*t* value	*p* uncorrected
*Pain > No-Pain*				
	*Left Accumbens*	*Trigeminal Nucleus*	2.13	0.038
	*Right Amygdala*	*Left Putamen*	2.11	0.040
	*Right Amygdala*	*Left Thalamus—S1*	2.10	0.041
	*Right Putamen*	*Right Thalamus—Dentate*	2.09	0.041
	*Left Caudate*	*Left Thalamus—Dentate*	2.02	0.048
*No-Pain > Pain*				
	*Right Caudate*	*Right Thalamus—M1*	3.28	0.002
	*Trigeminal Nucleus*	*Left Thalamus—S1*	2.73	0.009
	*Right Caudate*	*Right Thalamus—Dentate*	2.09	0.042
	*Left Accumbens*	*Left Thalamus—SMA*	2.09	0.042

All connections that were showing significance above *p* = 0.05 uncorrected are listed. No cluster wise correction was performed.

### Exploratory analysis of sub-groups

**Figure 2:**
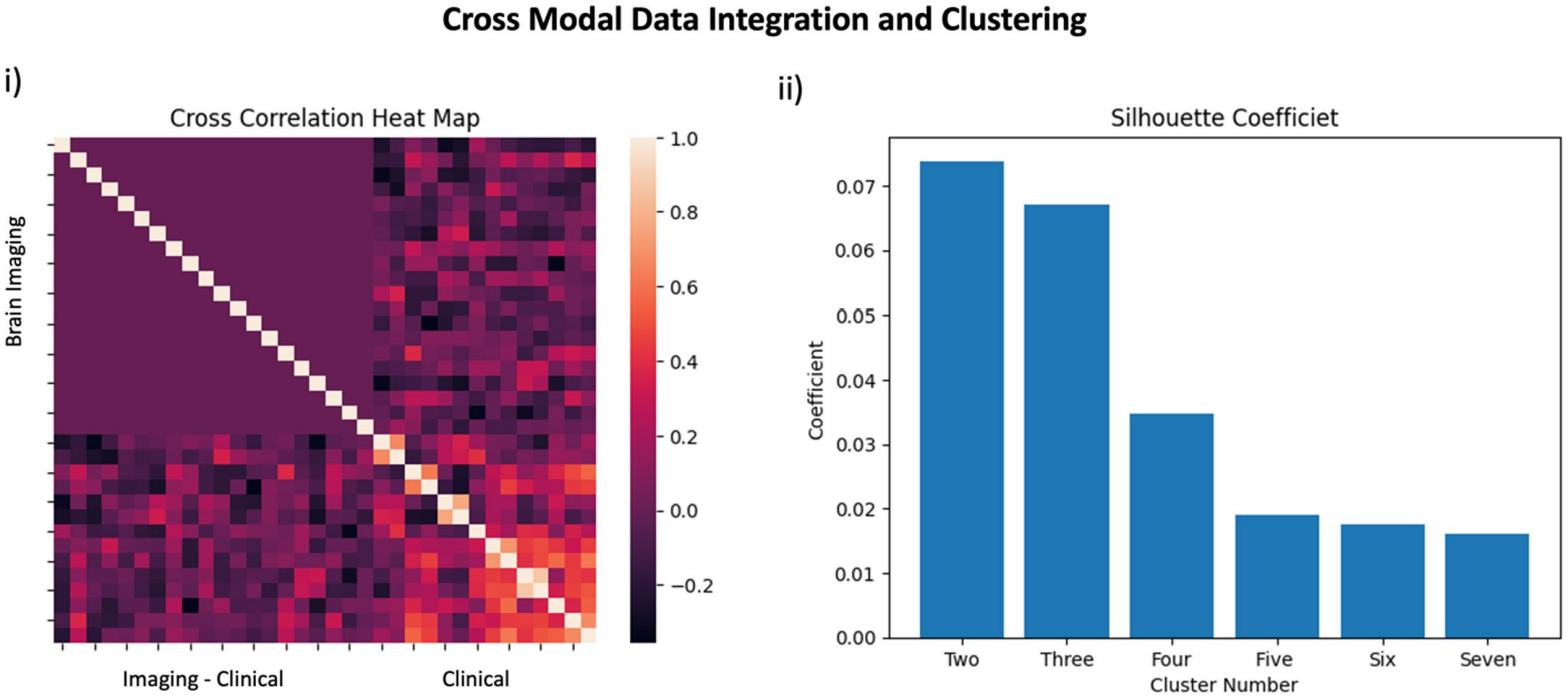
Data Integration: In panel **(i)** a cross correlation heatmap outlines the relationship between features included in the pain cohort analysis. The top left quadrant reflects brain imaging data whereas the bottom right quadrant reflects clinical data. The integration of brain imaging and clinical data is observed in the bottom left quadrant. In panel **(ii)**, silhouette coefficients are presented for use of two-through-seven clusters to support our use of two clusters in the pain cohort.

**Figure 3:**
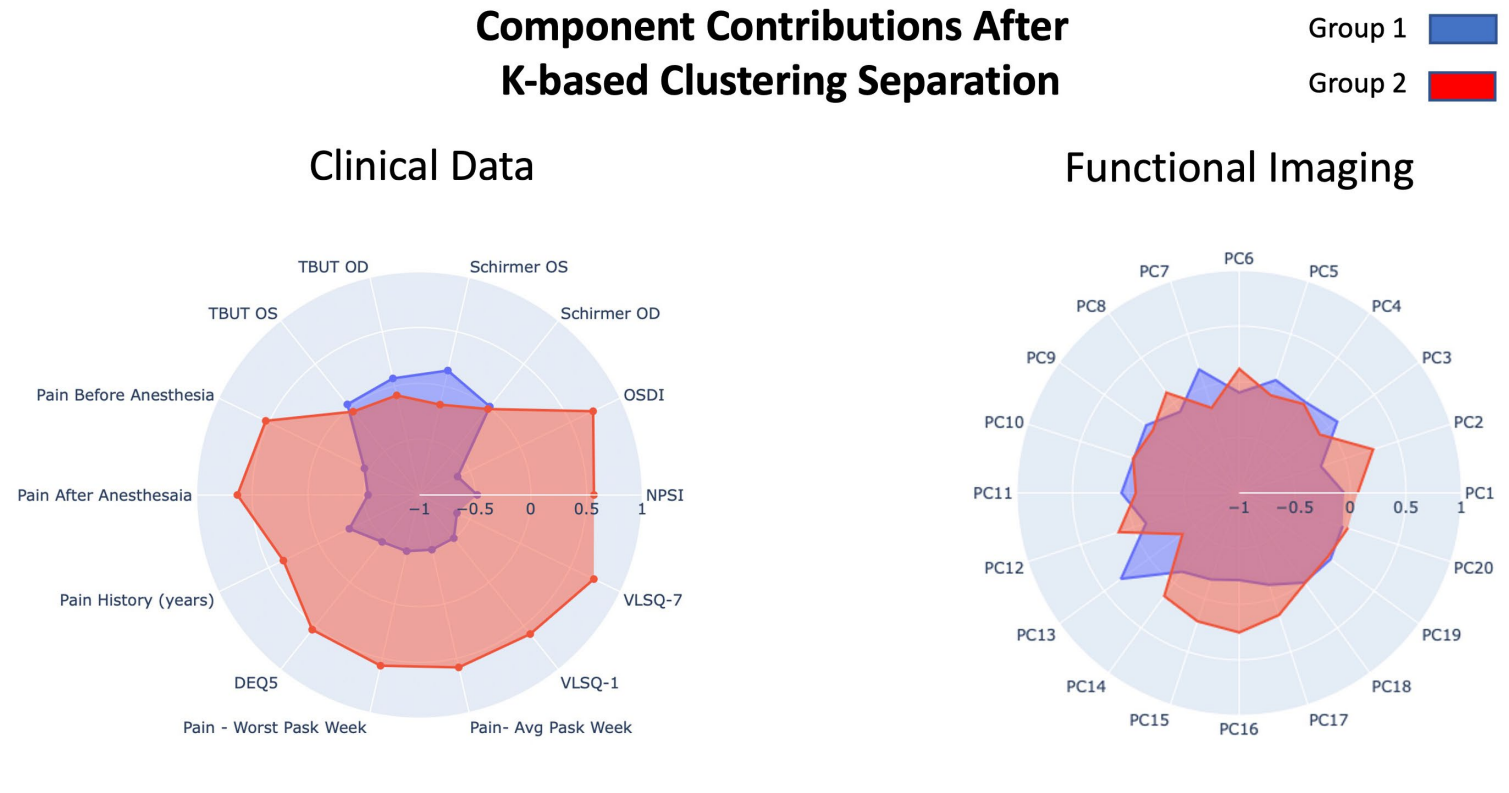
Overview of the results from the component loading after K-means clustering on our pain cohort. Resulting groups (Group 1 and Group 2) reflect division of the primary pain cohort. Clinical data (left) and functional imaging data (right) are presented in separate radar plots but were run in the same clustering analysis. The two outcome groups are presented in blue and red.

Prior to parsing our pain cohort into sub-cohorts, we integrated the functional brain imaging data with the clinical data to qualitatively evaluate the cross-correlation between constituent features. As can be observed in Figure 2i, the inter-correlation between clinical and of the functional neuroimaging data were variable, ranging from low to medium. Alternatively, inter-correlation between PC components of functional neuroimaging were weak (or absent)—supporting the success of the PCA analysis—and the clinical data showed moderate to strong inter-correlation values, as would be predicted from a thorough pain battery. We used a K-based clustering tool part of the Sklearn package and silhouette coefficients (Figure 2ii) to force data from our pain cohort (*n* = 37) into two cluster groupings. No participants from the non-pain cohort were included. All data was *z*-transformed prior to cluster analysis using the *z*-score function from scipy package in python. Groups were separated into two new pain cohorts with 17 individuals in one cohort and 20 in the other cohort. Outcomes reflecting clinical data and functional brain data are presented separately in Figure 3 using mean *z*-scores but were run in the same clustering analysis. Findings from the cluster analysis (compare blue and red overlays) suggest that there was greater division between cohorts on all clinical data, with exception of the TBUT and Schirmer scores that showed relatively minimal differences between cohorts. Clinically, these groups represent individuals with lower (Group 1) and higher (Group 2) levels of ocular pain with neuropathic features. The largest group divisions from the resting state fMRI data were found in the PC2, PC15, and PC16 components. Notably, these reflect latent component structures and not individual brain regions. We elected to show clinical scores as per the cluster division in Table 4 for clinical interpretation of our cluster findings.

**Table 4:** Descriptive statistics for the two pain groups produced from K-means clustering.

Referenced features	Group 1	Group 2
Years of DE diagnosis, mean ± SD	5.5 ± 6.44	9.71 ± 5.93
DEQ5 (range 0–22), mean ± SD	12.55 ± 3.05	15.71 ± 2.44
VLSQ-8-Q1 (range 0–4), mean ± SD	3.15 ± 1.31	4.53 ± 0.72
VLSQ-8-Q7 (range 0–4), mean ± SD	2.1 ± 1.25	4.11 ± 0.93
NPSI-Eye total (range 0–100), mean ± SD	27.75 ± 17.61	47.82 ± 15.81
OSDI total (range 0–100), mean ± SD	43.42 ± 15.51	72.13 ± 16.94
Worst pain elsewhere rating 1 week recall (range 0–10), mean ± SD^*^	4.5 ± 3.34	7.71 ± 1.49
Average pain elsewhere rating 1 week recall (range 0–10), mean ± SD	3.65 ± 3.07	6.76 ± 1.56
Ocular pain before anesthesia (mean ± SD; range 0–10)	1.9 ± 2.49	4.71 ± 2.64
Ocular pain after anesthesia (mean ± SD; range 0–10)^*^	0.9 ± 1.37	3.88 ± 2.75
Schirmer’s OD (mean ± SD; mm)	11.55 ± 9.11	11.29 ± 9.83
Schirmer’s OS (mean ± SD; mm)^**^	12.9 ± 11.89	9.94 ± 5.76
TBUT OS (mean ± SD; s)^*^	6.89 ± 4.71	6.28 ± 2.99
TBUT OS (mean ± SD; s)	7.81 ± 4.89	7.45 ± 3.75

These groupings reflect division of the primary pain cohort. We report the mean and standard deviation from the two pain clusters using original clinical scales (not *z*-scores) for ease of interpretation. *indicates this feature was ranked in the top five for differentiating our pain cohorts from our feature ranking analysis; ** indicates that this is the best performing feature from our list in differentiating our pain cohorts.

**Figure 4:**
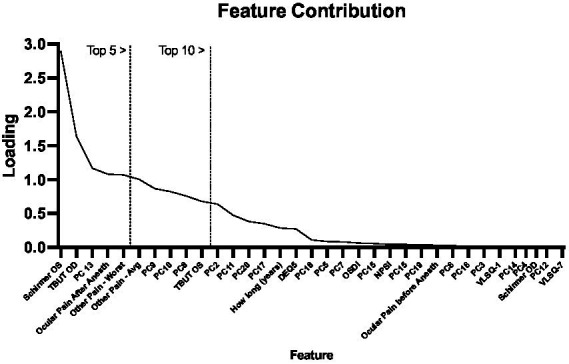
Feature contribution. Using the SelectKbest program, we show the relative contributions of each component to each pain group produced from the K-means Clustering approach. We outline using vertical lines the top 5 and 10 features.

We next ran the SelectKbest program for feature analysis to evaluate the feature contribution toward the cluster designations using the K-means clustering approach. This approach performs individual tests on each feature to determine which feature contributes most to the target variable (i.e., cluster designation). As shown in Figure 4, the top five features were the “Schirmer OS,” “TBUT OD,” “PC13,” and “Ocular pain—before and after anesthesia,” and “worst pain scores from the prior week.” This combination of features reflects objective clinical metrics (Schirmer and TBUT), self-report metrics (ocular pain reporting before and after anesthesia), and functional brain imaging (PC13) and suggests that even when considering pain and ocular surface metrics, a functional brain connectivity remains an important differentiator between pain cohorts.

## Discussion

To summarize the overall results of our study, we found that subcortical functional brain networks differ in individuals with chronic ocular pain and those without ocular pain symptoms. Beyond pain symptom reporting, elevated reporting was found in the pain cohort on metrics reflecting dry eye symptoms, and neuropathic pain, and photophobia. Observed differences in functional brain connectivity involved increased and decreased connectivity implying diverse network changes at the sub-cortical level. Lastly, through exploratory analyses, we provided evidence to suggest that behavioral and brain imaging metrics contribute to define sub-cohorts within the chronic ocular pain group, which may provide insight into the underlying mechanisms associated clinical metrics.

In this study, we found that persons with chronic ocular pain show evidence of neuropathic symptoms, including photophobia. These findings were observed when comparing our pain cohort relative to a group with no reporting of ocular pain, therein providing insight into comorbidities associated with ocular pain. A total of 31 persons in our pain cohort and nine persons in our control (no-pain) cohort had dry eye. Observing cases of dry eye in our control cohort may reflect sub-clinical pathology that is non-symptomatic, in line with prevalence of dry eye signs (35.2%) being higher than dry eye symptoms (11.59%) (34). We observed that both cohorts had similar demographics (including age) but trended toward a higher frequency of migraine in our pain cohort. This was not surprising given the close association between migraine and ocular pain noted in prior studies (25, 35). We observed that the pain cohort showed evidence of more ocular surface abnormalities, evidenced by tear break up time (TBUT) and Schirmer scores, however, the means in both populations were ≥ 5 s and 11 mm, respectively, the standard cut-offs for dry eye disease. In terms of self-reported pain scores, we showed that the eye pain cohort had neuropathic-like qualities as captured by the NPSI-Eye. Our pain cohort also reported photophobia symptoms, as queried by questions 1 and 7 on the VLSQ and question 9 of the NPSI-Eye. Additionally, we found that individuals in the pain group were more likely to demonstrate systemic sensitization to painful stimulation via the presence of 30-s aftersensations compared to the no-pain group (43 vs. 13%). Together, findings demonstrate that ocular pain from our included cohort is associated with neuropathic characteristics and reporting of centralized behaviors such as photophobia.

To further study, the impact of chronic ocular pain we examined brain connectivity and noted altered functional network connectivity between pain-relevant brain regions in persons reporting ocular pain versus not reporting ocular pain. Relative to the cohort without pain, those with pain showed increased functional connectivity—indicating regions that may be operating more in tandem—between the trigeminal nucleus and the accumbens which may relate to the role of the accumbens as a source of analgesia to mediate the presence of nociceptive signals (36) relayed from the innervation of the trigeminal nerve and ocular structures (37). In addition, those reporting pain showed increased connectivity between the putamen and left caudate, both implicated in pain detection (11, 38, 39), with the dentate gyrus sub-region of the thalamus. This aligns with prior work from our group, where we showed increased connectivity with hippocampal structures in persons with chronic pain (40), perhaps alluding to the consolidation of pain memories. Abnormalities in hippocampal structure (41, 42) and functional connectivity have been reported in prior work on chronic pain (43, 44) as well as persons with Sjögren’s Syndrome (45), supporting the role of hippocampal connectivity in the maintenance of ocular pain. A corollary decrease in connectivity—alluding to regions working less in tandem—between the dentate gyrus sub-region of the thalamus and caudate in the pain cohort may point toward hemispheric specialization of the hippocampus and preference of the left hippocampus for context-related, rather than spatial, memory (46). Observing multiple thalamic subregions having decreased connectivity in our pain cohort (see Table 2) may align with atrophy of the primary motor and sensory cortex as has been observed in chronic pain conditions (32). As such, we find evidence in our pain cohort for central nervous system changes that align with prior findings in other cohorts for a centralized impact of ocular pain.

A current lack in understanding of mechanisms that mediate the expression of ocular pain underscored our interests in using clinical, self-report, and radiological data in an integrated manner to group patients into clinical meaningful categories. We applied a K-means clustering technique to produce two groups from our pain cohort (participants from the no-pain group were excluded from this analysis). From a behavioral standpoint, there appeared to be separation between the two pain sub-cohorts—using average *z*-scores—on all metrics with what appeared to be less separation on the TBUT and Schirmer indices. Results were echoed with functional brain imaging data where group separation was observed on PC2, 13, 14, 15, 16, and 17, highlighting that our two pain cohorts could be dissociated based on mean (*z*-score) biobehavioral features. Notably, this was also observed when converted back to clinical scores (Table 4) and showcased that our two new pain sub-cohorts were largely differentiated into high and low clinical scores across each metric. After producing these two (high and low pain) cohorts, we elected to explore what features best differentiate these groups. Using a feature ranking algorithm, we found Schirmer, TBUT, and pain reporting contributed to the top five ranked features that distinguished our two pain cohorts in addition to PC13 from the fMRI data. This finding may appear confusing given that minimal differences are observed on clinical Schirmer’s OD scores; however, we believe that the success of this feature to differentiate pain cohorts is attributed to the unique variability between cohorts (Group 1 = 11.89; Group 2 = 5.76) relative to the cohort averages. Although tests such as NPSI had large differences between cohorts, they produced similar levels of variance. Moreover, broadly, shared variance between clinical metrics may have contributed to the redundancy of features and therefore a low ranking of feature importance for classification. To this point, it is notable that PC13, reflecting a latent feature of functional brain connectivity, was able to achieve high feature ranking status and aligns with prior work integrating brain and behavioral data for identifying pain cohorts (47). Though this analysis was exploratory, we believe these findings support future work aimed at identifying features to support the identification of pain phenotypes in persons with chronic ocular pain.

There are notable limitations requiring address in the current investigation. First, our no pain and pain cohorts represented a heterogeneous mixture of ocular participants. Based on recruitment procedures, we did not explicitly recruit our control sample to be absent of dry eye signs, as this is a common finding in the general population. As such, it is likely that peripheral abnormalities (tear instability, low tear production) differentially contributed to pain symptomatology in our pain cohort, and this may have confounded current analyses. As well, different ocular diseases will present with unique signs and symptoms, which may confound replication of study findings. Second, the current sample was biased toward inclusion of males. To date, we do not understand the unique physiological or behavioral components of male and female neuro-ophthalmological data. As such, this remains an item to be addressed in the coming investigations and should be kept in mind when interpreting findings. Lastly, current cohort sizes were relatively small. One area this may have direct relation to is our use of the preprocessing steps such as our spatial smoothing choice for image pre-processing. We recognize use of smaller filters (e.g., 4 mm) may address issues including artificial correlations; however, the choice to use an 8 mm was adopted to minimize the impact of noise in a relatively small sample cohort. We emphasize the exploratory nature of current findings as hypothesis generating and have used a conservative approach to discuss these findings. We hope that such analyses will guide larger cohort analyses in the future.

## Conclusion

Chronic ocular pain presents a clinical challenge by means of the presence and extent of ocular pathology, and the integration of centralized symptoms. The current investigation demonstrated that persons with chronic ocular pain exhibit broad pain behaviors that coincide with central functional network changes in subcortical brain regions implicated in pain perception. We show that further sub-typing is possible and should be explored in future work that integrates both clinical and neuroimaging data to understand symptoms such as photophobia and ocular pain when evidence of ocular surface abnormalities is present and absent.

## Data availability statement

The datasets presented in this article are not readily available because security and privacy rules regarding participants as part of Veterans Affairs. Requests to access the included datasets should be directed to eric.moulton@childrens.harvard.edu.

## Ethics statement

The studies involving humans were approved by Miami Veterans Affairs (VA) and the University of Miami Institution Review Boards (IRB approvals #3011.08 and 20190340, respectively). The studies were conducted in accordance with the local legislation and institutional requirements. The participants provided their written informed consent to participate in this study.

## Author contributions

SH: Conceptualization, Formal analysis, Writing – original draft, Writing – review & editing. NR: Data curation, Formal analysis, Methodology, Writing – review & editing. JH: Data curation, Methodology, Writing – review & editing. AG: Data curation, Funding acquisition, Methodology, Project administration, Resources, Supervision, Writing – review & editing. PP: Methodology, Writing – review & editing. EF: Funding acquisition, Methodology, Supervision, Writing – review & editing. EM: Conceptualization, Formal analysis, Funding acquisition, Investigation, Methodology, Supervision, Writing – review & editing.

## References

[1] Garza-León M Valencia-Garza M Martínez-Leal B Villarreal-Peña P Marcos-Abdala HG Cortéz-Guajardo AL. Prevalence of ocular surface disease symptoms and risk factors in group of university students in Monterrey, Mexico. J Ophthalmic Inflamm Infect. (2016) 6:44. doi: 10.1186/s12348-016-0114-z, PMID: 27864795 PMC5116015

[2] Skalicky SE Goldberg I McCluskey P. Ocular surface disease and quality of life in patients with Glaucoma. Am J Ophthalmol. (2012) 153:1–9.e2. doi: 10.1016/j.ajo.2011.05.033 21872203

[3] Kumar S Singh T Ichhpujani P Vohra S Thakur S. Correlation of ocular surface disease and quality of life in Indian Glaucoma patients: BAC-preserved versus BAC-free Travoprost. Turk J Ophthalmol. (2020) 50:75–81. doi: 10.4274/tjo.galenos.2019.29000, PMID: 32366084 PMC7204904

[4] Moshirfar M Benstead E Sorrentino P Tripathy K. Ocular Neuropathic Pain Stat Pearls Publishing (2023).

[5] Jacobs DS Lim M Carrasquillo KG Rosenthal P. Bevacizumab for corneal neovascularization. Ophthalmology. (2009) 116:592–3. doi: 10.1016/j.ophtha.2008.10.011 19264217

[6] Ong S Doraiswamy M Lad E. Controversie and future directions of ocular biomarkers in alzheimer disease. JAMA Neurol. (2018) 75:650–1. doi: 10.1001/jamaneurol.2018.0602, PMID: 29710250

[7] Basilious A Xu CY Malvankar-Mehta MS. Dry eye disease and psychiatric disorders: a systematic review and meta-analysis. Eur J Ophthalmol. (2022) 32:1872–89. doi: 10.1177/11206721211060963, PMID: 34935549 PMC9297048

[8] Galor A. How depression might relate to dry eye disease. JAMA Ophthamol. (2022) 140:399–400. doi: 10.1001/jamaophthalmol.2022.0146, PMID: 35266991 PMC13432165

[9] Crane AM Levitt RC Felix ER Sarantopoulos KD McClellan AL Galor A. Patients with more severe symptoms of neuropathic ocular pain report more frequent and severe chronic overlapping pain conditions and psychiatric disease. Br J Ophthalmol. (2017) 101:227–31. doi: 10.1136/bjophthalmol-2015-308214, PMID: 27130915 PMC5575758

[10] Rosenthal P Borsook D. The corneal pain system. Par 1: the missing piece of the dry eye puzzle. Ocul Surf. (2012) 10:2–14. doi: 10.1016/j.jtos.2012.01.002 22330055

[11] Borsook D Upadhyay J Chudler EH Becerra L. A key role of the basal ganglia in pain and analgesia-insights gained through human functional imaging. Mol Pain. (2010) 6:1744-8069-6-27. doi: 10.1186/1744-8069-6-27, PMID: 20465845 PMC2883978

[12] Galor A Levitt RC Felix ER Martin ER Sarantopoulos CD. Neuropathic ocular pain: an important yet underevaluated feature of dry eye. Eye. (2015) 29:301–12. doi: 10.1038/eye.2014.263, PMID: 25376119 PMC4366454

[13] Moulton EA Becerra L Borsook D. An fMRI case report of photophobia: activation of the trigeminal nociceptive pathway. Pain. (2009) 145:358–63. doi: 10.1016/j.pain.2009.07.018, PMID: 19674842 PMC2756998

[14] Choudhury A Reyes N Galor A Mehra D Felix E Moulton EA. Clinical neuroimaging of photophobia in individuals with chronic ocular surface pain. Am J Ophthalmol. (2023) 246:20–30. doi: 10.1016/j.ajo.2022.09.020, PMID: 36223850 PMC10964268

[15] Chen M-J Huang R Liang RB Pan YC Shu HY Liao XL. Abnormal intrinsic functional hubs in corneal ulcer: evidence from a voxel-wise degree centrality analysis. J Clin Med. (2022) 11:1478. doi: 10.3390/jcm11061478, PMID: 35329804 PMC8949159

[16] Tazi S Boulanouar AK Cassagne M Fournié P Malecaze J Payoux P. Abnormal brain function in photophobic patients with dry eye disease: an fMRI study. Rev Neurol (Paris). (2023) 179:599–606. doi: 10.1016/j.neurol.2022.11.014, PMID: 36863903

[17] Xu A Larsen B Baller EB Scott JC Sharma V Adebimpe A. Convergent neural representations of experimentally-induced acute pain in healthy volunteers: a large-scale fMRI meta-analysis. Neurosci Biobehav Rev. (2020) 112:300–23. doi: 10.1016/j.neubiorev.2020.01.004, PMID: 31954149 PMC7755074

[18] Pondelis NJ Moulton EA. Supraspinal mechanisms underlying ocular pain. Front Med. (2022) 8:768649. doi: 10.3389/fmed.2021.768649, PMID: 35211480 PMC8862711

[19] Li Y-Q Takada M Shinonaga Y Mizuno N. Direct projections from the midbrain periaqueductal gray and the dorsal raphe nucleus to the trigeminal sensory complex in the rat. Neuroscience. (1993) 54:431–43. doi: 10.1016/0306-4522(93)90264-G, PMID: 7687754

[20] Knight YE Goadsby PJ. The periaqueductal grey matter modulates trigeminovascular input: a role in migraine? Neuroscience. (2001) 106:793–800. doi: 10.1016/S0306-4522(01)00303-7, PMID: 11682164

[21] Belmonte C Acosta MC Merayo-Lloves J Gallar J. What causes eye pain? Curr Ophthalmol Rep. (2015) 3:111–21. doi: 10.1007/s40135-015-0073-9, PMID: 26000205 PMC4432221

[22] Borsook D Burstein R Becerra L. Functional imaging of the human trigeminal system: opportunities for new insights into pain processing in health and disease. J Neurobiol. (2004) 61:107–25. doi: 10.1002/neu.20085, PMID: 15362156

[23] Chalmers RL Begley CG Caffery B. Validation of the 5-item dry eye questionnaire (DEQ-5): discrimination across self-assessed severity and aqueous tear deficient dry eye diagnoses. Contact Lens Anterior Eye. (2010) 33:55–60. doi: 10.1016/j.clae.2009.12.010, PMID: 20093066

[24] Schiffman RM. Reliability and validity of the ocular surface disease index. Arch Ophthalmol. (2000) 118:615. doi: 10.1001/archopht.118.5.615 10815152

[25] Farhangi M Diel RJ Buse DC Huang AM Levitt RC Sarantopoulos CD. Individuals with migraine have a different dry eye symptom profile than individuals without migraine. Br J Ophthalmol. (2020) 104:260–4. doi: 10.1136/bjophthalmol-2018-313471, PMID: 31040130

[26] Verriotto JD Gonzalez A Aguilar MC Parel JMA Feuer WJ Smith AR. New methods for quantification of visual photosensitivity threshold and symptoms. Transl Vis Sci Technol. (2017) 6:18. doi: 10.1167/tvst.6.4.18, PMID: 28845363 PMC5566267

[27] Sall K Foulks GN Pucker AD Ice KL Zink RC Magrath G. Validation of a modified National eye Institute grading scale for corneal fluorescein staining. Clin Ophthalmol. (2023) 17:757–67. doi: 10.2147/OPTH.S398843, PMID: 36915716 PMC10007867

[28] Nieto-Castanon A Whitfield-Gabrieli S. Conn: a functional connectivity toolbox for correlated and anticorrelated brain networks. Brain Connect. (2012) 2:125–41. doi: 10.1089/brain.2012.0073 22642651

[29] Urien L Wang J. Top-down cortical control of acute and chronic pain. Psychosom Med. (2019) 81:851–8. doi: 10.1097/PSY.0000000000000744, PMID: 31609921 PMC6832828

[30] Yang S Chang MC. Chronic pain: structural and functional changes in brain structures and associated negative affective states. Int J Mol Sci. (2019) 20:3130. doi: 10.3390/ijms20133130, PMID: 31248061 PMC6650904

[31] Akram H. Dayal V. Mahlknecht P. Georgiev D. Hyam J. Foltynie T.. Connectivity derived thalamic segmentation in deep brain stimulation for tremor. NeuroImage Clin. (2018) 18:130–42. doi: 10.1016/j.nicl.2018.01.008 29387530 PMC5790021

[32] Holmes SA Barakat N Bhasin M Lopez NI Lebel A Zurakowski D. Biological and behavioral markers of pain following nerve injury in humans. Neurobiol Pain. (2020) 7:100038. doi: 10.1016/j.ynpai.2019.100038, PMID: 31890990 PMC6926375

[33] Pedregosa F. Varoquaux G. Gramfort A. Michel V. Thirion B. Grisel O.. Scikit=−learn: machine learning in Python. J Mach Learn Res. (2011) 12:2825–30.

[34] Papas E. The global prevalence of dry eye disease: a bayesian view. Ophthalmic Physiol Opt. (2021) 41:1254–66. doi: 10.1111/opo.12888, PMID: 34545606

[35] Baksh BS Garcia JC Galor A. Exploring the link between dry eye and migraine: from eye to brain. Eye Brain. (2021) 13:41–57. doi: 10.2147/EB.S234073, PMID: 33692643 PMC7939506

[36] Harris H Peng Y. Evidence and explanation for the involvement of the nucleus accumbens in pain processing. Neural Regen Res. (2020) 15:597–605. doi: 10.4103/1673-5374.266909, PMID: 31638081 PMC6975138

[37] Patel N Jozsa F Das J. Neuroanatomy, spinal trigeminal nucleus. In: Stat Pearls. Treasure Island (FL): Stat pearls publishing. (2023)

[38] Wunderlich AP Klug R Stuber G Landwehrmeyer B Weber F Freund W. Caudate nucleus and insular activation during a pain suppression paradigm comparing thermal and electrical stimulation. Open Neuroimaging J. (2011) 5:1–8. doi: 10.2174/1874440001105010001, PMID: 21643502 PMC3106353

[39] Starr CJ Sawaki L Wittenberg GF Burdette JH Oshiro Y Quevedo AS. The contribution of the putamen to sensory aspects of pain: insights from structural connectivity and brain lesions. Brain. (2011) 134:1987–2004. doi: 10.1093/brain/awr117, PMID: 21616963 PMC3122370

[40] Lemme J Holmes S Sibai D Mar'i J Simons LE Burstein R. Altered brain network connectivity underlies persistent post-traumatic headache following mild traumatic brain injury in youth. J Neurotrauma. (2021) 38:1632–41. doi: 10.1089/neu.2020.7189, PMID: 33183144 PMC8165471

[41] Berger SE Vachon-Presseau É Abdullah TB Baria AT Schnitzer TJ Apkarian AV. Hippocampal morphology mediates biased memories of chronic pain. Neuro Image. (2018) 166:86–98. doi: 10.1016/j.neuroimage.2017.10.030, PMID: 29080714 PMC5813825

[42] Apkarian AV Mutso AA Centeno MV Kan L Wu M Levinstein M. Role of adult hippocampal neurogenesis in persistent pain. Pain. (2016) 157:418–28. doi: 10.1097/j.pain.0000000000000332, PMID: 26313405 PMC4858177

[43] Mutso AA Petre B Huang L Baliki MN Torbey S Herrmann KM. Reorganization of hippocampal functional connectivity with transition to chronic back pain. J Neurophysiol. (2014) 111:1065–76. doi: 10.1152/jn.00611.2013, PMID: 24335219 PMC3949236

[44] Mutso AA Radzicki D Baliki MN Huang L Banisadr G Centeno MV. Abnormalities in hippocampal functioning with persistent pain. J Neurosci. (2012) 32:5747–56. doi: 10.1523/JNEUROSCI.0587-12.2012, PMID: 22539837 PMC3365570

[45] Zhang X-D Zhao LR Zhou JM Su YY Ke J Cheng Y. Altered hippocampal functional connectivity in primary Sjögren syndrome: a resting-state fMRI study. Lupus. (2020) 29:446–54. doi: 10.1177/0961203320908936, PMID: 32075510

[46] Burgess SE Gardell LR Ossipov MH Malan TP Jr Vanderah TW Lai J. Time-dependent descending facilitation from the rostral ventromedial medulla maintains, but does not initiate. Neuropathic Pain J Neurosci. (2002) 22:5129–36. doi: 10.1523/JNEUROSCI.22-12-05129.2002 PMID: 12077208 PMC6757729

[47] Holmes S Mar'i J Simons LE Zurakowski D LeBel AA O'Brien M. Integrated features for optimizing machine learning classifiers of pediatric and young adults with a post-traumatic headache from healthy controls. Front Pain Res. (2022) 3:859881. doi: 10.3389/fpain.2022.859881, PMID: 35655747 PMC9152124

